# Increasing standardized ileal digestible arginine to lysine ratio increases growth performance with limited effects on measured gut functions in 8.5- to 12-kg pigs

**DOI:** 10.1093/tas/txag161

**Published:** 2026-10-13

**Authors:** Marko Rudar, Alex C Outlaw, Paulo H A de Azevedo, Haejin Kim, David A Clizer, Keith Haydon, Jenna E Bayne, Samuel J Rochell

**Affiliations:** Department of Animal Science, Texas A&M University, College Station, TX 77843, United States; Department of Animal Sciences, Auburn University, Auburn, AL 36849, United States; Department of Animal Sciences, Auburn University, Auburn, AL 36849, United States; Department of Animal Sciences, Auburn University, Auburn, AL 36849, United States; CJ America Bio, Downers Grove, IL, United States; CJ America Bio, Downers Grove, IL, United States; Department of Clinical Sciences, College of Veterinary Medicine, Auburn University, Auburn, AL, United States; Department of Poultry Science, College of Agriculture, Auburn University, Auburn, AL, United States

**Keywords:** amino acid, citrulline, gut permeability, nitric oxide, swine, weaning

## Abstract

Limited Arg availability may impact growth performance in the early post-weaning period. Arginine, in addition to its main role as a building block for protein, is a substrate for nitric oxide production and polyamines, which could have implications for gut functionality in nursery pigs. The objective of this study was to determine the impact of increasing the ratio of dietary standardized ileal digestible (*SID*) Arg to Lys on nursery pig growth performance, nutrient digestibility, and gut function markers. A total of 240 pigs (initial body weight, 8.88 ± 1.15 kg) were weaned at 28 days and assigned to one of four dietary treatments (net energy, 2515 kcal/kg; crude protein, 22.7%; SID Lys, 1.34%; five mixed-sex pigs per pen): (1) R75 (SID Arg:Lys, 0.75; *n* = 12); (2) R90 (SID Arg:Lys, 0.90; *n* = 12); (3) R105 (SID Arg:Lys, 1.05; *n* = 12); and (4) R120 (SID Arg:Lys, 1.20; *n* = 12). Arginine, above that in the R75 diet, was supplied as crystalline L-Arg; diets were made isonitrogenous with L-Ala. Pigs had free access to diets for 14 d; body weight and pen feed disappearance were measured weekly and pen fecal samples were collected over the last 3 d of the study. At day 7 post-weaning, 24 pigs (*n* = 6 per treatment) were removed from the growth performance study and used for a parallel gut function study. A catheter was placed in the jugular vein for serial blood sampling. After recovering from surgery for 3 d, pigs were administered lactulose and mannitol to assess gut permeability and an intravenous injection of L-alanyl-L-glutamine to evaluate gut citrulline production. These pigs were subsequently euthanized and gut tissue was collected for morphology and digestive enzyme analysis. Increasing SID Arg:Lys ratio increased average daily gain, final body weight, and gain:feed ratio linearly (*P* < 0.05) without affecting average daily feed intake (*P* > 0.10). Apparent total tract digestible gross energy and crude protein also increased linearly (*P* < 0.05) and quadratically (*P* < 0.01) with increasing SID Arg:Lys ratio. Although increasing SID Arg:Lys ratio increased plasma nitric oxide concentration, SID Arg:Lys ratio did not affect gut morphology, digestive enzyme activity, permeability, and citrulline production. In summary, increasing SID Arg:Lys ratio up to 1.20 increases nursery pig growth performance in the early post-weaning period. The increase in performance, however, appears to be largely independent of measured impacts on gut function.

## Teaser

Nursery pig growth performance is maximized when the standardized ileal digestible Arg:Lys ratio is at least 1.20, but this response appears to be independent of any impact on gut health.

## Introduction

The immediate post-weaning period is associated with compromised gut health leading to poor growth performance, increased diarrhea incidence, and greater morbidity and mortality in pigs (Pluske 2016). In-feed antibiotics and pharmacological doses of zinc oxide help lessen the negative impacts of weaning stress on growth and enteric disease. However, antibiotic use for growth promotion in animal feed is banned due to concerns about antibiotic resistance (Casewell et al. 2003; FDA 2012; PHAC 2015). While pharmacological zinc oxide inclusion in nursery diets is still routine in the U.S., this practice has been banned in the European Union, and is being phased out in Canada, due to concerns about environmental heavy metal accumulation and development of antimicrobial resistance (Vahjen et al. 2015; Jensen et al. 2016). Since such regulations restrict the use of proven tools that promote growth performance and mitigate intestinal dysfunction in pigs at weaning, alternative and effective approaches are needed.

Functional amino acids are amino acids that exert physiological effects beyond their role as substrates for protein synthesis (Wu 2010). Among functional amino acids, Arg regulates protein synthesis through the mechanistic target of rapamycin complex signaling pathway (Chantranupong et al. 2016) and is a precursor for creatine, nitric oxide (*NO*), and polyamines (Wu et al. 2018); creatine is an energy buffer in skeletal muscle, NO promotes angiogenesis and stimulates vasodilation, and polyamines enhance intestinal epithelial cell proliferation. Endogenous Arg synthesis depends on coordinated intestinal and renal metabolism, where citrulline (*Cit*) generated by the small intestine is converted to Arg primarily in the kidneys (Brosnan and Brosnan 2004; Marini et al. 2017). While Arg is produced in the liver within the urea cycle, hepatic release of Arg is negligible due to rapid hydrolysis of Arg to urea and ornithine (*Orn*) (Deutz et al. 1998; Bruins et al. 2003). Arginine is considered a conditionally indispensable amino acid (*IAA*) in pigs and, under most conditions, endogenous synthesis is sufficient to meet requirements for growth and other physiological functions (Wu et al. 1997). However, it is possible that endogenous Arg synthesis is insufficient after weaning when poor feed intake and enteric disease can compromise gut function and, ostensibly, Arg production from gut-derived Cit.

The current estimated standardized ileal digestible (*SID*) Arg requirement for 8.5- to 12-kg pigs is approximately 3.4 g/d, corresponding to an SID Arg:Lys ratio of 0.46 (NRC 2012). Since corn- and soybean meal-based nursery diets typically provide more SID Arg than required by NRC (2012), resulting in an SID Arg:Lys ratio of at least 0.80, meeting Arg requirements is seldom considered in practical diet formulation. Yet, neither the NRC (2012) estimate nor current approaches to diet formulation account for the roles of Arg linked to gut function. Thus, establishing SID Arg requirements of weaned pigs and evaluating the necessity of synthetic Arg supplementation in current nursery diets is needed to support optimal growth performance and gut function. Increasing SID Arg intake above NRC (2012) recommendations, such that the SID Arg:Lys ratio is between 0.95 and 1.15, has been reported to enhance growth performance in nursery pigs (Hernandez et al. 2009; Yao et al. 2011; Hagen et al. 2024; Humphrey et al. 2024; Perez-Palencia et al. 2024). Detrimental impacts of excess Arg intake on growth performance have also been reported (Southern and Baker 1982; Hagemeier et al. 1983; Greiner et al. 2023). Further, relatively limited information is available on how Arg intake affects gut function during the immediate post-weaning period (Greiner et al. 2023; Hagen et al. 2024). Additional characterization of the relationship between Arg intake and indices of gut function may provide some insight into how Arg contributes to improved growth performance. The objective of this study was to determine the extent to which increasing SID Arg:Lys ratio affects growth performance, nutrient digestibility, and gut function markers in nursery pigs. We hypothesized that an SID Arg:Lys ratio above 0.90 will improve both nursery pig growth performance and gut function.

## Materials and methods

All animal procedures were approved by the Institutional Animal Care and Use Committee at Auburn University (PRN 2024–5423).

### Animals and housing

Pigs (Yorkshire × Duroc × Hampshire) were obtained from the Swine Research and Education Center at Auburn University. At 28 d age, a total of 240 pigs were weaned and divided into nursery room pens according to body weight (*BW*) and sex (five mixed-sex pigs per pen; 12 pens per four dietary treatments described below) across five replicate blocks. For experiment 1, all pigs were housed in the same nursery room of 18 pens (1.5 m × 2 m) with polypropylene flooring positioned above 36-cm deep pits that were flushed weekly for waste management; not all pen spaces were used per block. Pens were equipped with an adjustable single-sided five-hole nursery feeder (76 cm length × 29 cm width; AP-SN*305; Automated Production Systems, Sterling Heights, MI) and cup waterer (270650; QC Supply, Schuyler, NE). Initial room temperature was set to 29°C and was reduced by 1.7°C per week for 2 weeks. For experiment 2, pigs were housed individually in metabolism crates providing (7 sq. ft. per pig) in climate-controlled rooms.

### Animal diets

Four treatment diets were formulated to meet or exceed estimated requirements for all nutrients, except for Arg, for 8.5- to 12-kg pigs (NRC 2012). Diets were corn- and soybean meal-based (Table 1; net energy, 2,515 kcal/kg; crude protein [*CP*], 22.7%; SID Lys, 1.34%): R75 (SID Arg:Lys, 0.75); R90 (SID Arg:Lys, 0.90); R105 (SID Arg:Lys, 1.05); and R120 (SID Arg:Lys, 1.20). Corn distillers dried grains and solubles and corn gluten meal were included at the expense of soybean meal to reduce SID Arg content in the R75 diet. Arginine, above that in the R75 diet, was supplied as crystalline L-Arg; diets were made isonitrogenous with L-Ala. The R90 and R105 diets were prepared by blending appropriate amounts of the R75 and R120 diets together (R90, 2 parts R75 plus 1 part R120; R105, 1 part R75 plus 2 parts R120). Diets samples were analyzed for CP (method 990.03; AOAC Int., 2006) and total amino acids (method 982.30; AOAC Int., 2006) by the University of Missouri Agricultural Experiment Station Chemical Laboratories. Diets were provided to pigs as a mash.

**Table 1:** Ingredient composition and calculated nutrient content of treatment diets.

Item	Diet^a^	
Ingredient composition, %	R75	R120
**Corn**	44.94	45.53
**Corn DDGS**	10.00	10.00
**Soybean oil**	1.00	1.00
**Whey permeate**	15.00	15.00
**Soybean meal**	17.50	17.50
**Corn gluten meal**	4.00	4.00
**Spray-dried blood plasma**	2.50	2.50
**L-Lys•HCl**	0.56	0.56
**DL-Met**	0.13	0.13
**L-Thr**	0.13	0.13
**L-Trp**	0.02	0.02
**L-Val**	0.02	0.02
**L-Arg**	–	0.61
**L-Ala**	1.20	–
**Limestone**	1.40	1.40
**Monocalcium phosphate**	0.60	0.60
**Salt**	0.30	0.30
**Vitamin premix****^b^**	0.25	0.25
**Trace mineral premix****^c^**	0.15	0.15
**Titanium dioxide**	0.30	0.30
**Calculated nutrient content**		
**ME, kcal/kg**	3381	3381
**NE, kcal/kg**	2517	2513
**CP, %**	22.7	22.7
**SID Lys, %**	1.35	1.35
**SID Lys:ME, g/Mcal**	3.95	3.95
**SID Arg, %**	1.00	1.61
**SID** Arg:Lys	0.74	1.19
**Total Ca, %**	0.77	0.77
**STTD P, %**	0.38	0.38

a The R90 and R105 diets were prepared by blending appropriate amounts of the basal R75 and R120 diets together (R90, 2 parts R75 plus 1 part R120; R105, 1 part R75 plus 2 parts R120).

b Vitamin premix provided the following per kg complete diet: vitamin A, 6600 IU; vitamin D3, 1100 IU; vitamin E, 26 IU; vitamin K, 1.1 mg; vitamin B12, 28 μg; riboflavin, 6.0 mg; niacin, 27.5 mg; and pantothenic acid, 45.1 mg; and choline, 110 mg.

c Trace mineral premix provided the following per kg complete diet: Fe (as ferrous sulfate), 150 mg; Cu (as copper sulfate), 15 mg; Zn (as zinc sulfate), 150 mg; Mn (as manganous oxide), 40 mg; I (as calcium iodate), 0.5 mg; and Se (as sodium selenite), 0.3 mg.

Abbreviations used: CP, crude protein; ME, metabolizable energy; NE, net energy; SID, standardized ileal digestible; STTD, standardized total tract digestible.

### Experimental procedures and sample collection

#### Experiment 1

At 28 d age, pigs were weighed (initial BW, 8.88 ± 1.15 kg) and pens were assigned to one of four dietary treatments (*n* = 12 pens per diet): (1) R75; (2) R90; (3) R105; or (4) R120. Pigs were fed in a single phase for 14 d; feed addition per pen was weighed and recorded daily and pig BW and pen feed disappearance were measured and recorded weekly to calculate average daily gain (*ADG*), average daily feed intake (*ADFI*), and feed efficiency (*G:F*; gain-to-feed ratio). Feed and water were provided ad libitum. Pig morbidity, mortality, and removals were monitored daily. On day 14, feed was removed from each pen feeder with a wet/dry vacuum. After withholding access to feed for 2 h, the pig closest to the mean BW of the pen was selected and bled by jugular venipuncture. Blood (6 mL) was collected into di-potassium ethylenediaminetetraacetic acid (*EDTA*) vacutainer tubes (BD, Franklin Lakes, NJ). Plasma was separated from whole blood by centrifugation (3000 × g for 15 min at room temperature), aliquoted, and stored at −20°C until analysis.

Fecal samples were collected for apparent total tract digestibility (*ATTD*) of dry matter (*DM*), gross energy (*GE*), and CP and fecal consistency scores on days 11 through 14. Pens were cleaned prior to and every 24 h thereafter during fecal sample collection. Pens were monitored hourly between 08:00 and 18:00 and fecal samples (combination of fresh samples from the pen floor and fresh samples from rectal palpation) were collected, pooled per pen, and frozen at −20°C. Only fecal samples that were obtained by rectal palpation were assessed for fecal consistency. Fecal consistency from at least three pigs per pen was assessed on a five-point scale: 1 = liquid; 2 = soft, unformed feces that does not retain shape; 3 = soft, moist feces that retains shape; 4 = firm, formed stool; and 5 = hard, dry pellet. Fecal consistency data is reported as the proportion of pigs per pen with a score of 1 or 2.

#### Experiment 2

At 7 d post-weaning, 6 pigs from the second, third, fourth, and fifth replicates (24 pigs in total; *n* = 6 pigs per treatment) were weighed, removed from the growth performance study, and used for the gut health study. At the time of selection, pigs were confirmed to have been consuming treatment diets based on change in BW and visual observations. A single catheter (silicone; inner diameter, 1.58 mm; outer diameter, 2.41 mm; Freudenberg Medical, Carpinteria, CA) was placed surgically in the left external jugular vein per pig. Pigs were sedated midazolam (0.2 mg/kg intramuscular) and general anesthesia was induced with isoflurane (5% induction, 2% maintenance) after an overnight fast. The ventral neck was clipped, scrubbed, and cleaned with alternating washes of ethanol and betadine. Meloxicam (0.2 mg/kg subcutaneous) and lidocaine/bupivacaine (2%/0.5%; 0.5 mL at the incision site) were administered to pigs to provide general and local analgesia, respectively. Following incision, exposure of the vein, and catheter placement and securement, the catheter was tunneled to the ipsilateral shoulder (de Lange et al. 1989). The incision was closed in two layers; topical antibiotic ointment was applied to the incision and the neck was wrapped in gauze and bandage. Pigs were moved to metabolism crates once fully recovered from anesthesia and able to stand and move without assistance. Pigs were offered their assigned treatment diets within 3 h of recovery, which were provided ad libitum for an additional 3 d. Catheter function was checked daily.

On day 4, after a 6-h fast, pigs were administered an oral gavage of lactulose (500 mg/kg) and mannitol (50 mg/kg) to assess intestinal permeability and an intravenous injection of L-alanyl-L-glutamine (500 mg/kg) to assess gut Cit production with the citrulline generation test (CGT). Blood (2 mL) was collected from pigs into di-potassium EDTA vacutainer tubes before administration and at 15, 30, 45, 60, 90, 120, 180, 240, 300, and 360 min thereafter from the indwelling catheter. Following the end of serial blood collection, pigs were allowed free access to feed for 30 min. Pigs were euthanized by carbon dioxide asphyxiation 60 min after feed removal (ie 90 min after offering feed). A postprandial blood sample (6 mL) was collected into di-potassium EDTA vacutainer tubes just before euthanasia; jejunum (sampled 2.5 m after the ligament of Treitz) and ileum (sampled 20 cm before the ileocecal junction) was collected after euthanasia for histology and molecular analyses. Plasma was separated from whole blood by centrifugation (3000 × g for 15 min at room temperature), aliquoted, and stored at −20°C until analysis. For histology analysis, 5-cm segments of jejunum and ileum were rinsed with ice-cold phosphate-buffered saline and placed in neutral-buffered formalin. For molecular analyses, 20-cm segments of jejunum and ileum were opened longitudinally, rinsed with ice-cold phosphate-buffered saline, scaped with a microscope slide to separate the mucosa from the underlying serosa, snap-frozen in liquid nitrogen, and stored at −80°C.

### Sample processing and laboratory analysis

#### Apparent total tract digestibility of dry matter, gross energy, and crude protein

Fecal samples per pen were dried for 72 h at 60°C, ground in a coffee grinder, and passed through a 1-mm particle size sieve. Dried and ground treatment diets and fecal samples were analyzed for ash (method 942.05; AOAC Int., 2006), GE (Parr 6400 Automatic Isoperibol Calorimeter), and CP (method 990.03; AOAC Int., 2006). Titanium content in treatment diet and fecal ash was analyzed according Myers et al. (2004), with minor modifications.

#### Plasma amino acid concentrations

Plasma samples (100 µL) were spiked with 100 µL of internal standard (norvaline, 500 µmol/L), deproteinized with 400 µL acetonitrile plus 400 µL methanol, and incubated overnight at −20°C. After centrifugation (12,000 × g for 30 min at 4°C), 800 µL deproteinized plasma was transferred to a clean 2-mL microcentrifuge tube, dried under vacuum for 4 h, and reconstituted in 200 µL of ultrapure water. The reconstituted sample (10 µL) or calibration standard (10 µL) and sodium tetraborate (70 µL; 125 mmol/L, pH 9.3) were added to a clean 2-mL microcentrifuge tube. Amino acids were derivatized with 20 µL 6-aminoquinolyl-N-hydroxysuccinimidyl carbamate (3 mg/mL in acetonitrile; Cayman Chemical, Ann Arbor, MI) for 1 min at room temperature and for 10 min at 55°C. Derivatized amino acids were separated on a C18 analytical column (Waters Acquity UPLC BEH C18, 1.7 µm × 150 mm; Waters, Milford, MA) on an Acquity UPLC H-Class Plus System equipped with a quaternary pump, tunable ultraviolet detector, and Empower 3 software (Waters). Mobile phases were 0.1% formic acid in water (mobile phase A) and acetonitrile (mobile phase B). Separation was performed at a flow rate of 300 µL/min with the following gradient: 0 min, 0% B; 8 min, 4% B; 12 min, 10% B; 20 min, 20% B; 24 min, 80% B; 27 min, 80% B; 27.5 min, 0% B; 30 min, 0% B. Gradient changes were linear. The autosampler temperature was 20°C, the column temperature was 55°C, the injection volume was 1 µL, and the detection wavelength was 254 nm.

An external calibration curve was constructed containing all proteinogenic amino acids plus taurine, Cit, ornithine (*Orn*), and norvaline standards (2.5 µmol/L to 500 µmol/L). Regression equations for each amino acid were generated by weighted (1/x) linear regression analysis from peak areas with the regression procedure of SAS (version 9.4; SAS Institute, Cary, NC). The concentration of each amino acid in the plasma samples was calculated with the corresponding regression equation, corrected for recovery based on the norvaline internal standard, and adjusted for sample dilution. Plasma amino acids are reported in µmol/L.

#### Plasma total nitric oxide concentration

Due to the short biological half-life of NO, plasma NO concentration was determined indirectly as the sum of plasma nitrite and nitrate concentrations using a commercial kit (Nitrate/Nitrite Colorimetric Assay Kit; Cayman Chemical, Ann Arbor, MI). The intra-assay CV was 1.36%; the inter-assay was not determined since all plasma samples were analyzed in duplicate on the same plate.

#### Plasma lactulose and mannitol concentrations

Plasma samples obtained 60 min and 240 min after oral administration of D-lactulose and D-mannitol were analyzed to determine their respective plasma concentrations. Plasma samples (5 µL) were spiked with 5 µL of internal standard (D-raffinose, 5 µg/mL), deproteinized with 10 µL ultrapure water, 40 µL acetonitrile, and 40 µL methanol, and incubated on ice for 10 min. After centrifugation (12,000 × g for 30 min at 4°C), 100 µL deproteinized plasma was transferred to a clean 1.5-mL microcentrifuge tube. The plasma sample was re-extracted with an additional 10 µL ultrapure water, 20 µL acetonitrile, and 20 µL methanol, as described above; 50 µL of the deproteinized plasma was transferred to the same 1.5-mL tube. The plasma extract was centrifuged at 12,000 × g for an additional 30 min at 4°C and 100 µL was transferred to an autosampler vial.

Analysis was performed on a Vanquish Flex Binary UPLC system (Thermo Fisher Scientific, Waltham, MA) coupled with a quadrupole orbitrap mass spectrometer (Orbitrap Exploris 120, Thermo Fisher Scientific) and Xcalibur software (version 4.4.16.14). Chromatographic separation was performed on an amide analytical column (Waters XBridge BEH amide, 3.5 µm, 2.1 × 50 mm; Waters), modified from Gervasoni et al. (2018). Mobile phases were ammonium formate in water (2 mmol/L, adjusted to pH 9; mobile phase A) and acetonitrile (mobile phase B). Separation was performed at a flow rate of 400 µL/min with the following gradient: 0 min, 90% B; 3 min, 75% B; 3.5 min, 40% B; 4 min, 40% B; 4.5 min, 90% B; 8.5 min, 90% B. Gradient changes were linear. The column was maintained at 60°C to suppress carbohydrate mutarotation while samples were held at room temperature. The injection volume was 5 µL. Mass spectrometry was performed with electrospray ionization in negative mode using an EASY-IC internal calibration at the start of the run. The following mass spectrometry parameters were used: scan range, m/z 100–1000; resolution, 120,000; S-lens RF voltage, 70%; maximum ion injection time, automatic; spray voltage, 2100 V; ion transfer tube temperature, 250°C; vaporizer temperature, 200°C; mild trapping, enabled. The following ions were monitored and quantified: lactulose (retention time, 3.28 min), m/z 341.1089 plus 386.1139 (formate adduct); mannitol (retention time, 1.71 min), m/z 181.0718 plus 227.0772; and raffinose (retention time, 4.39 min), m/z 503.1617 plus 549.1672.

An external calibration curve containing D-lactulose, D-mannitol, and D-raffinose standards (0.1 µg/mL to 100 µg/mL) was constructed. Standards were analyzed as described previously for plasma samples. Regression equations for each sugar were generated by linear regression analysis from peak areas with the regression procedure of SAS (version 9.4; SAS Institute, Cary, NC). The concentration of each sugar in the plasma samples was calculated with the corresponding regression equation, corrected for recovery based on the D-raffinose internal standard, and adjusted for sample dilution. Plasma lactulose and mannitol concentrations are reported in µg/mL.

#### Jejunum and ileum morphology

Morphometric analysis was performed on formalin-fixed jejunum and ileum. Samples were embedded in paraffin, sectioned at 4 µm, stained with hematoxylin and eosin, and imaged with a VS200 Research Slide Scanner (Olympus Life Sciences, Tokyo, Japan). Villus height (*VH*) and crypt depth (*CD*) were determined in 15 adjacent and intact villus-crypt axes per pig using QuPath image analysis software (version 0.4.3; Bankhead et al. 2017).

#### Jejunum and ileum amino acid contents

Jejunum and ileum mucosa (100 mg) was homogenized in 5 mL of acetonitrile, methanol, and water (40:20:20 v/v) spiked with norvaline (500 µmol/L), and incubated overnight at −20°C. After centrifugation (3,000 × g for 30 min at 4°C), 1 mL deproteinized tissue was transferred to a clean 2-mL microcentrifuge tube, dried under vacuum for 4 h, and reconstituted in 200 µL of ultrapure water. The reconstituted tissue sample and calibration standard were subsequently processed as described above for plasma amino acids. The concentration of each amino acid in the tissue samples was calculated with the corresponding regression equation, corrected for recovery based on the norvaline internal standard, and adjusted for sample dilution and tissue weight. Tissue amino acids are reported in nmol/g wet tissue weight.

#### Jejunum and ileum disaccharidase activities

Approximately 50 mg of jejunum and ileum mucosa were homogenized in a Mini-bead beater 24 (BioSpec Products, Bartlesville, OK) in 1 mL homogenization buffer (pH 7.4) containing D-mannitol (50 mmol/L), 4-(2-hydroxyethy)-1-piperazineethanesulfonic acid (HEPES; 20 mmol/L), NP-40 (1% v/v), and protease inhibitor (1% v/v; P8340, Millipore Sigma, St. Louis, MO). Protein concentrations of mucosal homogenates were measured in duplicate using the bicinchoninic acid assay. Specific enzyme activities of maltase, lactase, and sucrase were measured according to Dahlqvist (1968) with minor modifications. Mucosal homogenate (50, 100, and 100 µL for maltase, lactase, and sucrase, respectively) was diluted up to 200 µL with additional homogenization buffer in a clean 2-mL microcentrifuge tube. After adding 200 µL of pre-heated substrate buffer (50 mmol/L maltose, lactose, or sucrose in 0.1 mol/L disodium maleate; pH 6.0), the reactions for maltase, lactase, and sucrase were carried out at 37°C for 30, 60, and 60 min, respectively, on a thermal mixer (Thermo Fisher Scientific, Waltham, MA). The reaction was stopped with 100 µL of ice-cold trichloroacetic acid (10% w/v) and centrifuged (12,000 × g for 10 min at 4°C). The supernatant was transferred to a clean 1.5-mL microcentrifuge tube. Glucose concentrations in the supernatant were determined using a glucose oxidase reagent kit (Pointe Scientific Glucose [Oxidase] Liquid Reagents, Pointe Scientific, Canton Township, MI) in a 96-well microplate format. Glucose concentrations were converted to nmol of glucose liberated per milligram of protein per minute (U). Maltase activity was corrected by dividing the calculated value by two due to the generation of two glucose per maltose.

### Calculations and statistical analysis

Data was analyzed using with SAS (version 9.4; SAS Institute, Cary, NC). For experiment 1, growth performance (initial BW, final BW, ADG, ADFI, and G:F), ATTD of DM, GE, and CP, and plasma amino acid concentrations were analyzed with the generalized linear and mixed model procedure in SAS with a normal distribution and identity link function. Fecal consistency score proportions were analyzed with a binomial distribution and logit link function. The statistical model included dietary treatment as a fixed effect and pen and replicate block as random effects; initial BW was included as a covariate in the model for final BW, ADG, ADFI, and G:F. Pen was considered the experimental unit. Orthogonal linear and quadratic contrasts were performed to assess dose effects of increasing SID Arg:Lys ratios on response variables for experiment 1. For experiment 2, all response variables were analyzed with the generalized linear and mixed model procedure in SAS with a normal distribution and identity link function, except when noted otherwise. The statistical model included dietary treatment as a fixed effect and pig and replicate block as random effects. Pig was considered the experimental unit. Plasma Cit area under the curve (*AUC*) was estimated in GraphPad Prism (version 10.4.2; GraphPad Software, Boston, MA) and analyzed in SAS, as described above. Orthogonal linear and quadratic contrasts were performed to assess dose effects of increasing SID Arg:Lys ratios on response variables for experiment 2. Time to peak plasma Cit concentration was assessed with the nonparametric one-way procedure in SAS using the Kruskal–Wallis test. For both experiments, the normality of residuals was evaluated with the Shapiro-Wilk test. Data are presented as least-squares means ± SEM. Differences among least-squares means were considered significant at *P* ≤ 0.05 and a trend at 0.05 < *P* ≤ 0.10.

## Results

Total diet CP and amino acid concentrations in the R75 and R120 diets are consistent with calculated contents (Table 2).

**Table 2:** Analyzed crude protein and total amino acid content (as-fed basis) of treatment diets.^a^

	Diet^b^			
Item	R75	R90	R105	R120
**CP, %**	22.9 (22.7)	23.1	23.1	23.3 (22.8)
**Total Lys, %**	1.43 (1.49)	1.52	1.55	1.51 (1.49)
**Total Arg, %**	1.12 (1.10)	1.24	1.46	1.60 (1.71)
**Total Ala, %**	2.33 (2.37)	1.81	1.69	1.40 (1.18)
**Total Met, %**	0.51 (0.47)	0.46	0.46	0.45 (0.47)
**Total Thr, %**	1.09 (0.97)	0.99	1.02	1.04 (0.97)
**Total Trp, %**	0.24 (0.26)	0.23	0.26	0.26 (0.26)
**Total Val, %**	1.09 (1.03)	1.03	1.07	1.11 (1.03)
**Total Ile, %**	0.93 (0.85)	0.87	0.92	0.94 (0.85)
**Total Leu, %**	2.10 (2.12)	2.03	2.12	2.21 (2.13)
**Total Phe, %**	1.05 (1.05)	1.00	1.03	1.07 (1.03)
**Total His, %**	0.55 (0.55)	0.52	0.53	0.55 (0.55)

a Values in parentheses indicate formulated values.

b R75, SID Arg:Lys = 0.75; R90; SID Arg:Lys = 0.90; R105, SID Arg:Lys, 1.05; R120, SID Arg:Lys, 1.20.

Abbreviations used: SID, standardized ileal digestible.

### Experiment 1

Initial BW did not differ among treatments (*P* > 0.10; Table 3). Average daily gain increased with increasing SID Arg:Lys ratio from 192 g/d in R75 pigs to 222 g/d in R120 pigs (linear, *P* < 0.01). Since ADFI was not different among treatments (*P* > 0.10), feed efficiency (G:F) increased with increasing SID Arg:Lys ratio from 0.49 in R75 pigs to 0.53 in R120 pigs (linear, *P* < 0.01). Final BW likewise increased with increasing SID Arg:Lys ratio (linear, *P* < 0.05). The proportion of pigs with soft stools or diarrhea tended to decline with increasing SID Arg:Lys ratio (linear, *P* < 0.10).

**Table 3:** Growth performance and fecal consistency of pigs fed diets with increasing SID Arg:Lys ratios (Exp. 1).

	Diet^a^				SEM^b^	*P*-value^c^		
	R75	R90	R105	R120		Treatment	Linear	Quadratic
***n***	12	12	12	12				
**Initial BW, kg**	8.83	8.80	8.79	9.13	0.36	0.866	0.544	0.584
**Final BW,^d^ kg**	11.45	11.39	11.73	11.88	0.33	0.047	0.011	0.443
**ADG, g/d**	192	195	214	222	22	0.061	0.009	0.783
**ADFI, g/d**	396	396	396	412	30	0.844	0.484	0.602
**G:F, g/g**	0.488	0.492	0.538	0.535	0.025	0.027	0.007	0.801
**FCS^d^**	0.54	0.54	0.35	0.40	0.07	0.149	0.058	0.684

a Pigs were fed treatment diets at weaning (28 d age) for 14 d; R75, SID Arg:Lys = 0.75; R90; SID Arg:Lys = 0.90; R105, SID Arg:Lys, 1.05; R120, SID Arg:Lys, 1.20.

b Maximum value for the standard error of the mean.

c Initial BW was used as a covariate for the statistical analysis of final BW, ADG, ADFI, and G:F.

d FCS: 1 = liquid; 2 = soft, unformed feces that does not retain shape; 3 = soft, moist feces that retains shape; 4 = firm, formed stool; and 5 = hard, dry pellet. Reported means represent the proportion of pigs with FCS of 1 or 2.

Abbreviations used: ADFI, average daily feed intake; ADG, average daily gain; BW, body weight; FCS, fecal consistency score; G:F, gain-to-feed ratio; SID, standardized ileal digestible.

Apparent total tract digestibility of DM, GE, and CP were all affected by treatment (*P* < 0.01; Table 4). Specifically, these values increased quadratically as SID Arg:Lys ratio increased (*P* < 0.01), such that there was a 1.7%, 11.0%, and 11.6% increase in the ATTD of DM, GE, and CP respectively from R75 to R90 with no further increase from R90 to R120.

**Table 4:** Apparent total tract nutrient digestibility in pigs fed diets with increasing SID Arg:Lys ratios (Exp. 1).

	Diet^a^				SEM^b^	*P*-value		
	R75	R90	R105	R120		Treatment	Linear	Quadratic
***n***	12	12	12	12				
**ATTD DM, %**	91.6	93.2	92.8	92.4	0.6	< 0.001	0.102	< 0.001
**ATTD GE, %**	64.5	71.6	70.7	69.6	2.4	< 0.001	0.001	< 0.001
**ATTD CP, %**	62.3	69.5	68.4	66.4	2.3	< 0.001	0.029	< 0.001

a Pigs were fed treatment diets at weaning (28 d age) for 14 d; R75, SID Arg:Lys = 0.75; R90; SID Arg:Lys = 0.90; R105, SID Arg:Lys, 1.05; R120, SID Arg:Lys, 1.20.

b Maximum value for the standard error of the mean.

Abbreviations used: ATTD, apparent total tract digestible; CP, crude protein; DM, dry matter; GE, gross energy; SID, standardized ileal digestible.

Plasma Arg concentration increased with increasing SID Arg:Lys ratio from 88 µmol/L in the R75 pigs to 135 µmol/L in the R120 pigs (linear, *P* < 0.01; Table 5). This corresponded to a linear increase in plasma Orn (*P* < 0.01), but not Ala or Cit (*P* > 0.10), concentrations. Conversely, plasma Lys concentration decreased with increasing SID Arg:Lys ratio from 80 µmol/L in the R75 pigs to 44 µmol/L in the R120 pigs (linear, *P* < 0.05).

**Table 5:** Plasma amino acid concentrations (µmol/L) in pigs fed diets with increasing SID Arg:Lys ratios on day 14 (Exp. 1).

	Diet^a^				SEM^b^	*P*-value		
Item	R75	R90	R105	R120		Diet	Linear	Quadratic
***n***	12	12	12	12				
**Ala**	681	661	600	623	41	0.436	0.174	0.573
**Arg**	88	111	104	134	10	0.012	0.003	0.773
**Asn**	69	74	70	73	6	0.922	0.772	0.967
**Asp**	19	19	17	16	2	0.695	0.262	0.710
**Cit**	65	65	61	67	4	0.731	0.843	0.443
**Gln**	445	477	443	448	29	0.558	0.776	0.478
**Glu**	285	268	285	248	39	0.818	0.514	0.747
**Gly**	1138	1041	1139	1024	87	0.618	0.496	0.906
**His**	69	71	75	79	11	0.539	0.151	0.875
**Ile**	76	82	77	79	7	0.793	0.792	0.648
**Leu**	229	233	228	238	14	0.921	0.660	0.804
**Lys**	80	64	64	44	20	0.120	0.024	0.805
**Met**	57	60	57	57	5	0.948	0.962	0.719
**Orn**	88	104	102	125	8	0.025	0.005	0.676
**Phe**	94	101	94	99	7	0.606	0.635	0.756
**Pro**	362	378	341	366	22	0.490	0.758	0.801
**Ser**	181	180	195	208	12	0.298	0.076	0.552
**Tau**	86	92	81	80	13	0.889	0.575	0.788
**Thr**	246	297	270	353	35	0.077	0.030	0.596
**Trp**	37	45	34	37	5	0.249	0.627	0.582
**Tyr**	143	142	140	140	15	0.997	0.836	0.999
**Val**	138	143	130	154	17	0.380	0.443	0.336

a Pigs were fed treatment diets at weaning (28 d age) for 14 d; R75, SID Arg:Lys = 0.75; R90; SID Arg:Lys = 0.90; R105, SID Arg:Lys, 1.05; R120, SID Arg:Lys, 1.20.

b Maximum value for the standard error of the mean.

Abbreviations used: Cit, citrulline; Orn, ornithine; Tau, taurine; SID, standardized ileal digestible.

### Experiment 2

All pigs recovered from surgery and resumed consuming treatment diets without complication. On the final study day, two pigs (one each from the R105 and R120 groups) were excluded from serial blood collections and subsequent analyses (ie plasma lactulose, mannitol, and citrulline concentrations) due to intermittent catheter failure.

Postprandial plasma Arg concentration increased as the SID Arg:Lys ratio increased from 144 µmol/L in the R75 pigs to 202 µmol/L in the R120 pigs (linear, *P* < 0.05; Table 6), whereas postprandial plasma Ala concentration decreased as the SID Arg:Lys ratio increased from 863 µmol/L in the R75 pigs to 595 µmol/L in the R120 pigs (linear, *P* < 0.05). The increase in plasma Arg corresponded to a linear increase in total plasma nitrate concentration (*P* < 0.05). However, postprandial plasma Cit, Lys, and Orn concentrations were not different among groups (*P* > 0.10). Jejunum mucosa Ala and Lys content tended to decline with increasing SID Arg:Lys ratio (linear, *P* < 0.10; Table S1, available as supplementary data at *Translational Animal Science* online). In the ileum mucosa, only Ala content tended to decline with increasing SID Arg:Lys ratio (linear, *P* < 0.10; Table S2, available as supplementary data at *Translational Animal Science* online). Arginine content in both the jejunum and ileum mucosa was not affected by treatment (*P* > 0.10).

**Table 6:** Postprandial plasma total nitrate (µmol/L) and amino acid (µmol/L) concentrations in pigs fed diets with increasing SID Arg:Lys ratios (Exp. 2).

	Diet^a^				SEM^b^	*P*-value		
Item	R75	R90	R105	R120		Diet	Linear	Quadratic
***n***	6	6	5	5				
**Nitrate**	54	64	109	90	15	0.048	0.025	0.313
**Ala**	863	637	624	595	87	0.110	0.042	0.251
**Arg**	144	146	165	202	21	0.183	0.043	0.380
**Asn**	74	60	68	61	7	0.408	0.317	0.646
**Asp**	16	11	9	12	2	0.170	0.151	0.093
**Cit**	54	50	51	57	8	0.898	0.769	0.486
**Gln**	659	553	557	646	61	0.440	0.897	0.115
**Glu**	163	152	137	175	30	0.827	0.874	0.400
**Gly**	700	675	723	721	60	0.923	0.668	0.844
**His**	93	79	89	89	6	0.383	0.916	0.266
**Ile**	89	73	108	89	9	0.069	0.356	0.905
**Leu**	170	146	179	155	16	0.456	0.861	0.984
**Lys**	226	169	177	184	27	0.380	0.314	0.226
**Met**	57	51	43	46	8	0.624	0.261	0.582
**Orn**	92	85	101	129	21	0.453	0.172	0.386
**Phe**	106	95	109	101	7	0.487	0.972	0.785
**Pro**	311	272	299	315	26	0.607	0.732	0.290
**Ser**	136	112	107	95	15	0.251	0.060	0.671
**Tau**	52	46	37	44	7	0.468	0.283	0.342
**Thr**	211	155	171	169	20	0.195	0.215	0.167
**Trp**	50	39	44	43	5	0.428	0.483	0.320
**Tyr**	95	74	78	73	12	0.504	0.245	0.520
**Val**	207	164	234	198	13	0.008	0.470	0.798

a Pigs were fed treatment diets at weaning (28 d age) for 10 d (7 d pre-surgery, 3 d post-surgery); R75, SID Arg:Lys = 0.75; R90; SID Arg:Lys = 0.90; R105, SID Arg:Lys, 1.05; R120, SID Arg:Lys, 1.20.

b Maximum value for the standard error of the mean.

Abbreviations used: Cit, citrulline; Orn, ornithine; Tau, taurine; SID, standardized ileal digestible.

Jejunum VH, CD, and VH:CD ratio was not different among treatments (*P* > 0.10; Table 7). While ileum VH was also not different among treatments, ileum CD declined and VH:CD ratio increased as SID Arg:Lys ratio increased (linear, *P* < 0.05). While there were no overall treatment differences in maltase, sucrase, and lactase activities in the jejunum mucosa (*P* > 0.10; Table 8), maltase activity tended to increase in the R90 pigs compared to other pigs (*P* < 0.10).

**Table 7:** Jejunum and ileum morphology in pigs fed diets with increasing SID Arg:Lys ratios (Exp. 2).

	Diet^a^				SEM^b^	*P*-value		
	R75	R90	R105	R120		Diet	Linear	Quadratic
***n^c^***	5	4	4	5				
**Jejunum**								
**VH**	374	395	397	390	27	0.856	0.571	0.533
**CD**	244	214	234	213	13	0.201	0.176	0.725
**VH:CD**	1.58	1.89	1.77	1.89	0.15	0.172	0.109	0.422
**Ileum**								
**VH**	305	279	313	300	21	0.698	0.825	0.762
**CD**	227	195	206	181	9	0.100	0.038	0.793
**VH:CD**	1.41	1.46	1.59	1.73	0.09	0.052	0.008	0.596

a Pigs were fed treatment diets at weaning (28 d age) for 10 d (7 d pre-surgery, 3 d post-surgery); R75, SID Arg:Lys = 0.75; R90; SID Arg:Lys = 0.90; R105, SID Arg:Lys, 1.05; R120, SID Arg:Lys, 1.20.

b Maximum value for the standard error of the mean.

c Six pigs were excluded from morphometric analysis due to improper sample collection.

Abbreviations used: VH, villus height; CD, crypt depth; SID, standardized ileal digestible.

**Table 8:** Gut disaccharidase enzyme activity (nmol glucose•mg^−1^•min^−1^) in pigs fed diets with increasing SID Arg:Lys ratios (Exp. 2).

	Diet^a^				SEM^b^	*P*-value		
Item	R75	R90	R105	R120		Diet	Linear	Quadratic
***n***	6	6	6	6				
**Jejunum**								
**Maltase**	75.9	120.3	85.3	89.1	13.7	0.065	0.926	0.097
**Sucrase**	38.4	32.7	33.5	35.1	8.6	0.943	0.775	0.616
**Lactase**	27.4	27.1	29.3	21.4	4.7	0.553	0.384	0.368
**Ileum**								
**Maltase**	52.2	61.7	50.7	63.4	8.7	0.649	0.565	0.858
**Sucrase**	42.6	55.3	37.4	39.5	9.8	0.447	0.476	0.541
**Lactase**	1.3	1.4	2.1	1.6	0.4	0.346	0.291	0.374

a Pigs were fed treatment diets at weaning (28 d age) for 10 d (7 d pre-surgery, 3 d post-surgery); R75, SID Arg:Lys = 0.75; R90; SID Arg:Lys = 0.90; R105, SID Arg:Lys, 1.05; R120, SID Arg:Lys, 1.20.

b Maximum value for the standard error of the mean.

Abbreviations used: SID, standardized ileal digestible.

Plasma lactulose and mannitol concentrations and plasma lactulose:mannitol ratios were not different among treatments at 60 min or at 240 min post-challenge (*P* > 0.10; Table 9). Plasma lactulose concentration (*P* < 0.05) and plasma lactulose:mannitol ratios (*P* < 0.01) declined from 60 min to 240 min post-challenge.

**Table 9:** Plasma lactulose and mannitol concentrations (µg/mL) and lactulose:mannitol ratio in pigs fed diets with increasing ratios of SID Arg to SID lys (Exp. 2).

	Diet^a^				SEM^b^	*P*-value		
Item	R75	R90	R105	R120		Diet	Linear	Quadratic
***n***	6	6	5	5				
**60 min**								
**Lactulose**	7.4	10.8	9.4	9.0	1.7	0.375	0.615	0.208
**Mannitol**	18.4	25.7	19.8	24.0	2.9	0.058	0.264	0.466
**L:M^c^**	0.78	0.78	0.88	0.80	0.17	0.958	0.819	0.796
**240 min**								
**Lactulose**	6.9	6.7	8.4	5.5	1.0	0.257	0.541	0.167
**Mannitol**	26.0	23.7	26.1	21.3	3.8	0.677	0.410	0.700
**L:M^c^**	0.51	0.56	0.58	0.52	0.06	0.700	0.831	0.270

a Pigs were fed treatment diets at weaning (28 d age) for 10 d (7 d pre-surgery, 3 d post-surgery); R75, SID Arg:Lys = 0.75; R90; SID Arg:Lys = 0.90; R105, SID Arg:Lys, 1.05; R120, SID Arg:Lys, 1.20.

b Maximum value for the standard error of the mean.

c Lactulose:mannitol ratio was calculated by converting lactulose and mannitol to their respective molar concentration with the corresponding molecular weight of each sugar (lactulose, 342.3 g/mol; mannitol, 182.2 g/mol).

Abbreviations used: L:M, lactulose-to-mannitol ratio; SID, standardized ileal digestible.

Following the intravenous injection of L-alanyl-L-glutamine, there was a rapid rise, peaking 15 min post-injection, and an extended decline in plasma Ala (Fig. 1A**)** and Gln (Fig. 1B) concentrations over 6 h. Plasma Cit concentrations increased from a baseline of approximately 41 µmol/L to a peak of 58 µmol/L between 60 min and 120 min (*P* < 0.01; Fig. 1C). Plasma Arg (Fig. 1D) and Orn (Fig. 1E) concentrations also increased following the L-alanyl-L-glutamine injection (*P* < 0.01), but the duration of the rise was limited compared to Cit. While there was no difference in peak plasma Cit concentrations, time to peak plasma Cit concentrations, or plasma Cit AUC among treatments (*P* > 0.10), plasma Cit AUC tended to decrease from the R75 pigs to the R120 pigs (linear and quadratic; *P* < 0.10; Fig. 1F).

**Figure 1:**
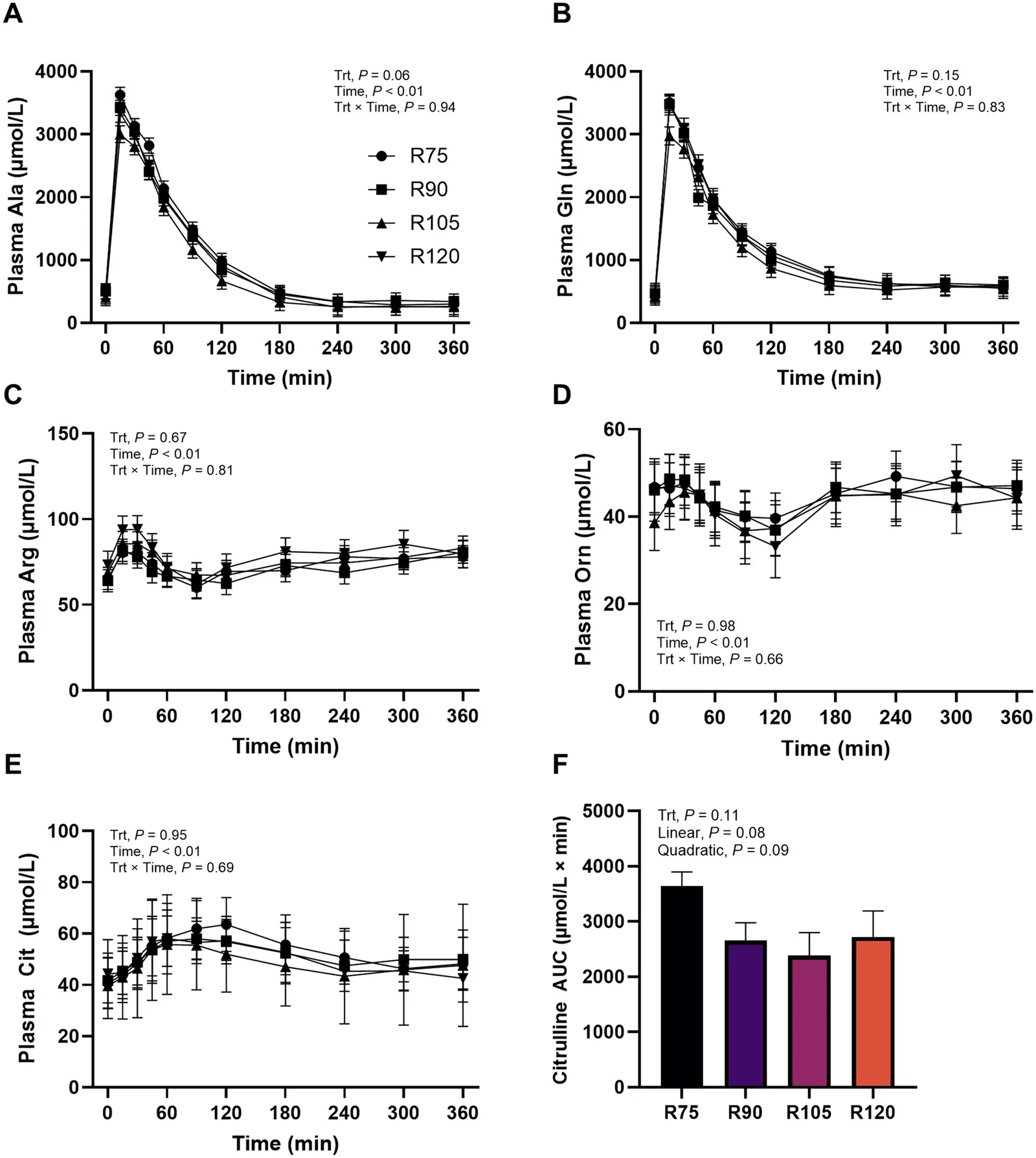
Plasma Ala (a), Gln (B), Arg (C), Cit (D), and Orn (E) concentrations and plasma Cit concentration AUC (F) over 6 h following an intravenous injection of L-alanyl-L-glutamine dipeptide (500 mg/kg) in nursery pigs fed diets with increasing SID Arg:Lys ratios (Experiment 2). Treatments: R75, SID Arg:Lys = 0.75 (*n* = 6); R90; SID Arg:Lys = 0.90 (*n* = 6); R105, SID Arg:Lys, 1.05 (*n* = 5); R120, SID Arg:Lys, 1.20 (*n* = 5). Values are least-squares means ± standard error of the mean. Abbreviations used: AUC, area under the curve; Cit, citrulline; Orn, ornithine; SID, standardized ileal digestible.

## Discussion

Arginine is a functional amino acid that is both a substrate for protein synthesis and precursor of creatine, NO, and polyamines (Wu et al. 2018). Considering that feed intake is typically low in the immediate postweaning period, Arg intake in newly weaned pigs is likely insufficient for growth and other metabolic demands. Further, an age-dependent decline in endogenous Arg synthesis suggests that Arg production may not fully close the gap between Arg supply and demand (Wu 1997). This suggests that increased dietary supply of Arg may be necessary in nursery diets. The current NRC (2012) recommendation for Arg is based on Southern and Baker (1983) who demonstrated that nursery pig growth performance increased with bioavailable Arg up to 0.48%, with no additional improvement at 0.58% and 0.68%. However, the SID Arg content of conventional corn- and soybean meal-based diets will often exceed 1%, implying that the NRC (2012) recommendations do not reflect current nursery feeding practices. The current experiment was carried out to evaluate the impact of increasing SID Arg:Lys ratios, from 0.75 to 1.20, on nursery pig growth performance, nutrient digestibility, and gut function. Among dietary IAA, Lys was supplied at the lowest level relative to estimated requirements for 8.5- to 12-kg pigs, ensuring accurate evaluation of Arg levels relative to Lys. Pharmacological zinc oxide was excluded from the treatment diets to avoid masking any potential benefits of additional Arg on measurements of intestinal function.

Feeding diets with increasing SID Arg:Lys ratios improved ADG and G:F without affecting ADFI, resulting in 0.43 kg greater BW at the end of the study for pigs provided SID Arg:Lys at 1.20 compared to 0.75. This corresponds to a SID Arg intake of 3.96 g/d at a SID Arg:Lys of 0.75 and 6.71 g/d at a SID Arg:Lys of 1.20. The SID Arg intake observed to maximize BW gain in the current study exceeded that reported by Greiner et al. (2023) by 0.70 g/d; however, considering that ADFI exhibited a quadratic response to increasing dietary SID Arg content, ADG and final BW were maximized at the highest SID Arg intake rather than the highest SID Arg content evaluated (Greiner et al. 2023). These levels of SID Arg intake are equivalent to SID Arg:Lys ratios of 1.03 and 1.09 for week 1 and weeks 2 and 3 after weaning, respectively. Perez-Palencia et al. (2024) reported that nursery pig growth performance is optimal when dietary SID Arg is supplied from 1.5% to 1.9%, corresponding to SID Arg:Lys ratios between 1.06 and 1.15 in phase 1 nursery diets and 1.10 and 1.34 in phase 2 nursery diets. Hagen et al. (2024) similarly reported a linear increase in nursery pig final BW as SID Arg:Lys ratios increased from 0.85 to 1.15. While these values exceed those reported by Humphrey et al. (2024), who estimated SID Arg:Lys ratios of 0.96 and 0.97 to maximize ADG and ADFI, respectively, in 6- to 13-kg nursery pigs, they are still consistent with the minimum SID Arg:Lys ratio of 0.81 estimated by the same authors for this BW range. The absence an ADFI response with increasing SID Arg:Lys ratio in the current study is in contrast with Greiner et al. (2023) and Humphrey et al. (2024) but is consistent with Hagen et al. (2024) and Perez-Palencia et al. (2024), suggesting that variation in feed intake can be attributed to factors other than Arg, at least when Arg is not provided in substantial excess (Southern and Baker 1982). In summary, optimal SID Arg:Lys ratios in nursery pigs generally range from 0.95 to 1.15 across studies, depending on age, health status, diet complexity, and endpoint evaluated. The present findings suggest that newly weaned pigs may respond to even greater ratios in the immediate post-weaning period.

As expected, increasing Arg intake resulted in dose-dependent increases in plasma Arg concentrations. This was paired with decreases in plasma Ala concentration in Experiment 2 but not in Experiment 1. The discrepancy in plasma Ala concentrations can be attributed to an interaction between hepatic Ala metabolism and time of blood sampling. Despite substantial liver uptake of Ala (Bruins et al. 2003), some diet-derived Ala escapes hepatic metabolism and enters systemic circulation; this is more likely to be the case in pigs consuming a large meal following a 12-h fast (ie in Exp. 1) than in non-fasted pigs consuming smaller meals throughout the day (ie in Exp. 2). Conversely, hepatic uptake of Arg is minimal compared to Ala (Bruins et al. 2003; Mansilla et al. 2018). Increased systemic Arg availability in turn increased both plasma Orn and total nitrate (ie NO) concentrations. Ornithine, derived from Arg hydrolysis, is the direct precursor for polyamine synthesis and, although not measured in the current study, polyamines are thought to regulate intestinal barrier integrity and stimulate gut development (Bekebrede et al. 2020). Nitric oxide, also derived from Arg, could promote blood flow through skeletal muscle and the gut to support nutrient delivery and waste removal and enhance immune cell function (Wu and Morris 1998); however, circulating NO concentrations do not provide insight into the site of NO production. Lastly, the decline in plasma Lys concentration with increasing SID Arg:Lys ratio (Exp. 1) could suggest that Arg, as another basic amino acid, interfered with intestinal Lys absorption. However, the lack of a difference in plasma His concentration and increase in growth performance as the SID Arg:Lys ratio increased indicates that is unlikely. While Perez-Palencia et al. (2024) reported that plasma Lys levels decreased with increasing Arg intake, other IAA levels also decreased. In this case, the decline in plasma IAA levels and increase in growth performance with increasing SID Arg intake implies greater overall IAA utilization for protein accretion. A key difference between Perez-Palencia et al. (2024) and the current study is the minimum SID Arg concentration evaluated. In Perez-Palencia et al. (2024), much of the reduction in plasma Lys and other IAA, likely reflecting greater incorporation into protein, occurred between an Arg-limiting diet (0.66% SID Arg) and an Arg-marginal diet (0.96% SID Arg), with the latter being most comparable to the lowest SID Arg content used in the current study (1.00% SID Arg). Subsequent increases in SID Arg intake above 1.00% would be expected to produce more modest changes in plasma AA concentrations. Indeed, Perez-Palencia et al. (2024) reported no changes in plasma IAA concentrations, apart from Arg, in a separate experiment in which dietary SID Arg content increased from 1.15% to 2.35% even though pig growth performance improved.

Despite the growing consensus that increasing Arg intake promotes growth performance in nursery pigs (Hernandez et al. 2009; Greiner et al. 2023; Hagen et al. 2024; Humphrey et al. 2024; Perez-Palencia et al. 2024), most recent studies have not reported its effects on nutrient digestibility. In the current study, increasing the SID Arg:Lys ratio was associated with greater ATTD of DM, GE, and CP. It should be noted, however, that the greatest increase in nutrient digestibility occurred when SID Arg:Lys ratio increased from 0.75 to 0.90, with no further benefit beyond 0.90, suggesting a minimum ratio between 0.75 and 0.90 to maximize nutrient digestibility. While increased nutrient digestibility, paired with smaller proportion of pigs with loose stools, could contribute to the greater growth performance with increasing SID Arg:Lys ratio, these differences did not correspond to substantial differences in jejunum and ileum villus height or to differences in disaccharidase enzyme activity. Greiner et al. (2023) and Hagen et al. (2024) also did not find changes in gut morphology with increasing Arg intake. Thus, it is unclear exactly how greater Arg intake improved nutrient digestibility. Arginine supplementation has been reported to upregulate the expression of specific amino acid transport systems in growing pigs (Morales et al. 2021). This, together with increased plasma NO concentrations, greater intestinal mucosa blood flow, and removal of absorbed nutrients from enterocytes, could point toward increased absorptive efficiency. Arginine, however, does not consistently enhance blood flow through the gut (Puiman et al. 2011). Alternatively, Arg supplementation has been reported to reduce basal endogenous losses of amino acids in growing pigs under heat stress conditions (Morales et al. 2023). Reduced enterocyte turnover in the ileum, suggested by shorter crypts with increasing SID Arg:Lys ratio, is consistent with this possibility and would inflate ATTD of CP.

The dual sugar absorption test is considered a reliable indicator of intestinal permeability in humans (Sequeira et al. 2014). Briefly, lactulose is absorbed via the paracellular pathway, and its absorption into systemic circulation increases when gut barrier function is compromised. In contrast, mannitol is absorbed via the transcellular pathway, and its uptake into systemic circulation is largely independent of gut barrier function. Although urine is typically collected from humans during the dual sugar absorption test, Hu et al. (2023) recently demonstrated that plasma and urine lactulose:mannitol ratios were highly correlated in pigs and, accordingly, that the plasma lactulose:mannitol ratio could provide insight on intestinal permeability in pigs (Wilson et al. 2022; Wensley et al. 2023; White et al. 2024). In the current study, the plasma lactulose:mannitol ratio was not different among treatments at both 60 min and 240 min following the sugar gavage, suggesting comparable gut barrier integrity in pigs across SID Arg:Lys ratios. While the ratio declined from 60 min to 240 min, this is unlikely to reflect any meaningful change in intestinal permeability and more likely reflects less lactulose absorption from the gut and cumulative lactulose excretion in urine. This finding is consistent with Greiner et al. (2023) and Hagen et al. (2024) who reported that serum lactulose:mannitol ratios were not affected by increasing Arg intake at either one or three weeks after weaning. The high variability of the dual sugar absorption test may prevent widespread use in such nutrition studies in which anticipated changes in gut barrier function are relatively small.

Considering that Cit is produced mainly in enterocytes, is usually absent in the diet, and largely escapes hepatic metabolism, plasma Cit concentration has been proposed as a marker for gut mass and function in pigs after weaning (Sciascia and Metges 2023). We previously demonstrated that acute feed restriction in weaned pigs lowers plasma Cit concentrations compared with Cit in age-matched and sow-reared pigs, corresponding to shorter jejunum and ileum villi heights (Gachman et al. 2024). Berkeveld et al. (2008) similarly reported reductions in plasma Cit concentrations on days 4 and 7 in conventionally weaned pigs compared to pigs that remained with the sow. In the current study, plasma Cit concentrations were not different among treatments in either experiment, indicating that gut function was similar across a range of SID Arg:Lys ratios. Since a single plasma Cit concentration may not fully reflect gut mass and function, a CGT was additionally performed. In the CGT, Gln is administered as a bolus dose in fasted pigs via the dipeptide L-alanyl-L-glutamine and the subsequent increase in plasma Cit concentration is measured (Peters et al. 2008). In the current study, peak plasma Cit concentrations, time to peak plasma Cit concentration, and plasma Cit AUC among treatments were not different among treatments, reinforcing comparable gut function in pigs across SID Arg:Lys ratios. While the trend for decreasing plasma Cit AUC with increasing SID Arg:Lys is difficult to explain, it is possible that pigs compensated for low Arg intake with greater Cit production, and subsequent Arg production, from Gln. Indeed, plasma Arg concentration was similar among groups during the CGT despite later postprandial differences. This would imply Arg-dependent regulation of enzymes involved in Cit synthesis, including as ornithine aminotransferase, carbamoyl phosphate synthase 1, and ornithine transcarbamylase. Available evidence indicates that intestinal expression of these enzymes in pigs is regulated primarily by age rather than Arg intake (Marini et al. 2017; Mohammad et al. 2020), but studies directly examining dietary regulation by Arg are lacking. Collectively, these findings suggest that plasma Cit concentrations and the CGT may not be sufficiently sensitive to detect modest diet-induced changes in gut function but can discriminate more substantial differences, such as those between weaned and non-weaned pigs or between healthy pigs and pigs with compromised intestinal function from enteric disease.

In conclusion, growth performance in 8.5- to 12-kg newly weaned pigs is greatest when the dietary SID Arg:Lys ratio is at least 1.20. This ratio corresponds to dietary SID Arg content of 1.61% and is in line with other recent estimates. However, no plateau was observed, suggesting that the SID Arg:Lys ratio needed to maximize growth performance may be greater than 1.20. Despite changes in circulating plasma Arg, Orn, and NO concentrations, the observed improvement in growth performance appeared to be largely independent of gut function; weaning age, which increased developmental maturity of the gut (Moeser et al. 2017), and sample size may have limited out ability to detect more subtle effects of Arg intake on intestinal function. Overall, this work suggests that the practical SID Arg:Lys requirement of nursery pigs is greater than current recommendations. Further research that further refines the SID Arg:Lys requirement, defines an upper limit to dietary Arg inclusion, and evaluates Arg-induced changes in plasma and gut polyamine status are warranted.

## Supplementary Material

txag161_Supplementary_Data

## List of abbreviations

ADG: average daily gain
ADFI: average daily feed intake
ATTD: apparent total tract digestible
BW: body weight
CD: crypt depth
CGT: citrulline generation test
Cit: citrulline
CP: crude protein
DAA: dispensable amino acid
DM: dry matter
EDTA: ethylenediaminetetraacetic acid
GE: gross energy
G:F: gain-to-feed
IAA: indispensable amino acid
NE: net energy
NO: nitric oxide
Orn: ornithine
SID: standardized ileal digestible
VH: villus height
