# Supplementary material for: Increasing standardized ileal digestible arginine to lysine ratio increases growth performance with limited effects on measured gut functions in 8.5- to 12-kg pigs
Source: Transl Anim Sci. 2026 Oct 13;10:txag161. doi: 10.1093/tas/txag161 (PMC13716491; doi:10.1093/tas/txag161)
Supplement: Supplementary file 1 [file txag161_supplementary_data.docx]

**Supplementary Table 1.** Jejunum free amino acid concentrations (nmol/g) in pigs fed diets with increasing SID Arg: Lys ratios (Exp. 2).

|  | Diet^1^ | | | | SEM^2^ | P-value | | |
| --- | --- | --- | --- | --- | --- | --- | --- | --- |
| Item | R75 | R90 | R105 | R120 |  | Diet | Linear | Quadratic |
| *n* | 6 | 6 | 6 | 6 |  |  |  |  |
| Ala | 2848 | 3106 | 2369 | 2449 | 265 | 0.110 | 0.073 | 0.708 |
| Arg | 413 | 567 | 472 | 421 | 85 | 0.268 | 0.781 | 0.106 |
| Asn | 532 | 636 | 491 | 507 | 98 | 0.391 | 0.447 | 0.514 |
| Asp | 1382 | 1474 | 1461 | 1368 | 146 | 0.936 | 0.934 | 0.532 |
| Cit | 188 | 209 | 147 | 165 | 35 | 0.282 | 0.223 | 0.938 |
| Gln | 1244 | 1522 | 1249 | 1437 | 147 | 0.169 | 0.506 | 0.675 |
| Glu | 5603 | 5993 | 5893 | 6036 | 385 | 0.856 | 0.495 | 0.752 |
| Gly | 4785 | 5238 | 4982 | 4948 | 456 | 0.916 | 0.909 | 0.599 |
| His | 209 | 297 | 194 | 209 | 29 | 0.076 | 0.430 | 0.216 |
| Ile | 298 | 440 | 348 | 335 | 84 | 0.382 | 0.939 | 0.209 |
| Leu | 592 | 905 | 722 | 684 | 183 | 0.361 | 0.864 | 0.180 |
| Lys | 411 | 497 | 373 | 336 | 48 | 0.107 | 0.098 | 0.196 |
| Met | 170 | 206 | 180 | 170 | 27 | 0.592 | 0.794 | 0.301 |
| Orn | 129 | 131 | 104 | 121 | 16 | 0.579 | 0.437 | 0.631 |
| Phe | 286 | 427 | 341 | 324 | 77 | 0.320 | 0.909 | 0.163 |
| Pro | 818 | 1104 | 882 | 901 | 140 | 0.249 | 0.950 | 0.216 |
| Ser | 841 | 1103 | 722 | 851 | 164 | 0.067 | 0.419 | 0.512 |
| Tau | 2993 | 2946 | 2944 | 3107 | 293 | 0.974 | 0.792 | 0.718 |
| Thr | 586 | 729 | 582 | 559 | 102 | 0.305 | 0.454 | 0.252 |
| Trp | 79 | 111 | 83 | 83 | 18 | 0.308 | 0.787 | 0.238 |
| Tyr | 248 | 356 | 280 | 261 | 69 | 0.392 | 0.858 | 0.206 |
| Val | 438 | 610 | 496 | 476 | 102 | 0.386 | 0.998 | 0.206 |

^1^ Pigs were fed treatment diets at weaning (28 d age) for 10 d (7 d pre-surgery, 3 d post-surgery); R75, SID Arg: Lys = 0.75; R90; SID Arg: Lys = 0.90; R105, SID Arg: Lys, 1.05; R120, SID Arg: Lys, 1.20.

^2^ Maximum value for the standard error of the mean.

Abbreviations used: Cit, citrulline; Orn, ornithine; Tau, taurine; SID, standardized ileal digestible.

**Supplementary Table 2.** Ileum free amino acid concentrations (nmol/g) in pigs fed diets with increasing SID Arg: Lys ratios (Exp. 2).

|  | Diet^1^ | | | | SEM^2^ | P-value | | |
| --- | --- | --- | --- | --- | --- | --- | --- | --- |
| Item | R75 | R90 | R105 | R120 |  | Diet | Linear | Quadratic |
| *n* | 6 | 6 | 6 | 6 |  |  |  |  |
| Ala | 2749 | 2665 | 2223 | 2251 | 241 | 0.303 | 0.087 | 0.818 |
| Arg | 345 | 382 | 406 | 392 | 53 | 0.825 | 0.445 | 0.605 |
| Asn | 488 | 449 | 342 | 395 | 63 | 0.404 | 0.187 | 0.473 |
| Asp | 2030 | 1923 | 1699 | 1889 | 192 | 0.676 | 0.460 | 0.450 |
| Cit | 185 | 196 | 186 | 173 | 42 | 0.967 | 0.740 | 0.721 |
| Gln | 1514 | 1286 | 1278 | 1533 | 150 | 0.474 | 0.940 | 0.123 |
| Glu | 8250 | 8268 | 7469 | 7761 | 673 | 0.787 | 0.451 | 0.838 |
| Gly | 5301 | 6519 | 5699 | 5924 | 557 | 0.428 | 0.652 | 0.356 |
| His | 251 | 247 | 235 | 225 | 23 | 0.847 | 0.387 | 0.893 |
| Ile | 275 | 277 | 262 | 243 | 30 | 0.813 | 0.382 | 0.716 |
| Leu | 536 | 575 | 528 | 494 | 62 | 0.798 | 0.507 | 0.534 |
| Lys | 376 | 313 | 377 | 339 | 54 | 0.723 | 0.828 | 0.790 |
| Met | 149 | 137 | 130 | 123 | 13 | 0.299 | 0.065 | 0.825 |
| Orn | 119 | 112 | 154 | 156 | 23 | 0.376 | 0.136 | 0.833 |
| Phe | 261 | 269 | 270 | 241 | 25 | 0.813 | 0.591 | 0.446 |
| Pro | 1049 | 1075 | 982 | 908 | 124 | 0.781 | 0.363 | 0.692 |
| Ser | 824 | 733 | 554 | 725 | 101 | 0.323 | 0.305 | 0.212 |
| Tau | 2310 | 2473 | 2456 | 2380 | 229 | 0.953 | 0.848 | 0.602 |
| Thr | 656 | 630 | 583 | 528 | 59 | 0.445 | 0.114 | 0.809 |
| Trp | 77 | 73 | 73 | 64 | 9 | 0.641 | 0.246 | 0.747 |
| Tyr | 241 | 237 | 226 | 203 | 27 | 0.711 | 0.282 | 0.698 |
| Val | 432 | 437 | 425 | 389 | 44 | 0.830 | 0.442 | 0.620 |

^1^ Pigs were fed treatment diets at weaning (28 d age) for 10 d (7 d pre-surgery, 3 d post-surgery); R75, SID Arg: Lys = 0.75; R90; SID Arg: Lys = 0.90; R105, SID Arg: Lys, 1.05; R120, SID Arg: Lys, 1.20.

^2^ Maximum value for the standard error of the mean.

Abbreviations used: Cit, citrulline; Orn, ornithine; Tau, taurine; SID, standardized ileal digestible.
